# Live fish highway: Uncovering the pathways that move millions of minnows across the United States

**DOI:** 10.1371/journal.pone.0347150

**Published:** 2026-05-13

**Authors:** Victoria DeRooy, Amanda Hansen

**Affiliations:** Upstream Aquatic Institute, Dover, Delaware, United States of America; Mansoura University, EGYPT

## Abstract

The spread of aquatic invasive species and pathogens over the last few decades has disrupted sportfishing, imposed economic costs on local industries, and threatened freshwater ecosystems. A significant but unquantified pathway for the spread of aquatic invasive species and pathogens in the United States is the long-distance transport of live baitfish. Despite the large scale of American baitfish production, policymakers lack high-quality, quantitative data on the movement of these fish across state lines, hindering rigorous risk assessment and regulatory efforts. This study addresses this knowledge gap by systematically collecting and synthesizing data on the interstate trade of live freshwater baitfish, obtained using freedom-of-information requests lodged with state governments across the United States. Our dataset captured the cross-border transport of 39.3 million live baitfish between 2022–24 in addition to 746 transport events where fish quantity was not recorded. Using this data, we detail trade flows disaggregated by species and explore the importance of key source states like Arkansas, South Dakota, and Minnesota. We highlight the variability in state government record-keeping, with data gaps existing in some states. By identifying high-traffic trade routes and quantifying species data, this study provides actionable insights for state agencies to strengthen biosecurity and environmental policy.

## Introduction

A risk to sportfishing, ecological sustainability, and water-based industries and economies around the world is the spread of aquatic invasive species [1–4]. One topic of interest is the movement of live fish for use as bait by recreational anglers [5]. Management strategies to prevent the spread of aquatic invasive species — species introduced outside of their native range which cause negative impacts in their introduced range — and pathogens via the live baitfish trade vary significantly by region. For example, Canadian provinces vary from the approach of Ontario, which uses Bait Management Zones to regulate the movement of live baitfish, to the approach of British Columbia and Alberta, which do not permit the use of live baitfish [6]. Likewise in the European Union, live baitfish is permitted in Czechia and prohibited in the Netherlands [7,8]. The economic costs associated with the spread of aquatic invasive species can be substantial [9,10]. According to the evidence systematically compiled in the InvaCost database of invasive species, the median economic cost associated with an aquatic invasive species in the United States is around 7.4 million USD [11,12]. Beyond economic value, aquatic invasive species may hinder the provision of ecosystem services that are intrinsically valued by society [13].

However, the United States offers a valuable focal point for research due to the unique commercial scale of its live baitfish trade. Unlike other countries that rely primarily on wild-harvested baitfish, the U.S. has a large, specialized baitfish aquaculture industry. The long-distance trade in live baitfish is notable for its scale in terms of both quantity of fish and geographic reach. While there are only 205 farms producing live baitfish in the United States according to the 2023 Census of Aquaculture, these farms are often industrialized enterprises producing large quantities of fish; in 2023, these farms collectively produced over one billion baitfish [14–16]. The live baitfish industry was responsible for 48 million USD of revenue in 2023 and contributes to states’ recreational fishing and outdoor industries [5,15]. The number of farms is a decrease from the 257 farms active in 2005, but an increase from the 168 farms active in 2018 [15,17]. Anecdotal evidence has suggested that the distributor markets may be undergoing consolidation, and there has also been suggestion of general market decline in some areas [5]. If there is indeed a widespread industry trend towards consolidation and lower sales, this could potentially reduce monitoring costs due to having fewer actors involved in the supply chain.

A number of regulations and initiatives seek to reduce the risk of aquatic invasive species and pathogens in the American live baitfish trade, though this varies by state. Many importing states (e.g., Nebraska, New York, Ohio, and others) require annual health testing of source aquaculture facilities, particularly for viral haemorrhagic septicaemia and occasionally for other pathogens, and major producers also conduct biosurveillance for some specific aquatic invasive species and pathogens [18–20]. Other states (e.g., Maine, Arizona) regulate the movement of live baitfish and/or the use of live baitfish in sensitive ecosystem areas [18,19].

Despite the scale and geographic reach of this flow of fish across the United States, neither the Federal nor state governments publish statistics on the interstate movement of live baitfish. Without such data, policymakers cannot rigorously assess the number of fish entering a given jurisdiction (e.g., a state) or the origin of those fish, essential information for quantifying the risk presented by this pathway of aquatic invasive species and pathogens and for crafting regulations to protect recreational fisheries, commercial fisheries, and local biodiversity. Therefore, there is a need for a comprehensive dataset on the interstate movement of live baitfish across the United States.

Several studies have provided initial insight into the trade of live baitfish among U.S. states. Some of these have focused on baitfish imports into a single state, limiting their applicability [21,22]. Van Senten and Engle [14] surveyed baitfish and sportfish producers in 13 U.S. states and found that 70% of respondents ship live fish to other states. On average, respondents reported shipping fish to 10 destination states. While this confirms the geographic reach and complexity of the baitfish trade, that survey did not identify the destination states. Snyder et al [23] surveyed bait shops and asked about the sources of bait, including state of origin. That study revealed baitfish flows between Michigan, Ohio, Arkansas, and Indiana. However, that study’s scope was limited due to its focus on the Great Lakes states and the fact that not all bait shops chose to divulge the identities of their suppliers. Gunderson [5] surveyed industry representatives of 28 states and found that many states imported live baitfish, especially from Arkansas, Minnesota, South Dakota, and Wisconsin. However, that study was limited to the states located in the Mississippi River Basin, and the author did not quantify these trade flows. Finally, Meronek et al [24] did quantify trade flows for a handful of importing states, though the data from that study is now over 30 years old and only covers six states.

As such, no study has provided quantitative, country-wide data on the interstate flow of live baitfish in the United States, a significant knowledge gap that limits the ability of policymakers and other stakeholders to rigorously assess the risk posed by this pathway of invasive species and pathogens. In this study, we fill that knowledge gap by systematically collecting and synthesizing data on the live freshwater baitfish trade from across the United States.

## Method

To obtain data on the interstate transport of live baitfish, we lodged a freedom-of-information/open records request with most U.S. state governments.

We lodged a request with every state government, except those states where live fish cannot be used as bait in freshwater (Alaska, Idaho, Oregon, Utah, and Washington) and those states where live baitfish cannot be imported for use in freshwater *and* live baitfish are not farmed in-state (Hawaii and Montana) [18]. For the remaining states, we determined the government department or agency responsible for governing imports and, if live baitfish are farmed in-state, the government department or agency responsible for governing aquaculture. This resulted in 58 government departments across 43 states.

For each of those 58 state government departments, we lodged a freedom-of-information/open records request with that department under the corresponding state’s freedom-of-information/open records legislation. The request letter asked for copies of the completed license application documents corresponding to all licenses granted for the import of live finfish into the state during calendar years 2022, 2023, and 2024, as well as additional documents held by the department that contains details of species and quantity of live fish imported into the state during that period (full request text is available as S1 Text). We also followed up several times with the handful of departments that did not promptly acknowledge the request, official acknowledgement within a period of several days being standard practice and/or a statutory requirement in most states. In some cases, the department sought clarification of the scope of the request, which we provided.

In 16 states, departments either confirmed that the requested records did not exist or provided records that exclusively related to non-bait purposes (Table 1). Six requests were rejected for reasons of in-state citizenship requirements, which exist in four states — this corresponded to one request for each of Alabama and Kentucky and two requests for each of Arkansas and Tennessee. A further two requests were not answered even after multiple follow-ups. We followed up at least twice with each state and waited a minimum of 6 months before excluding a state. A handful of states also held records for only some groups of live baitfish. For example, South Carolina only maintained records for trout used as live baitfish, though these trout records did not enable us to trace the origin of the trout.

**Table 1:** States that prohibit live baitfish importation/use or that did not keep records.

State prohibits live baitfish importation or use	Washington, Oregon, Idaho, Utah, Alaska, Montana, Maine, Minnesota
State did not keep import records	New York, New Jersey, Missouri, Georgia, Kentucky, Maine, Mississippi, Missouri, North Carolina, Ohio, Delaware, Wisconsin, Minnesota, Mississippi, South Carolina, Michigan, Florida

Upon receipt of the requested documents in PDF, XLS and/or DOCX file formats, we manually parsed the documents to create a standardized spreadsheet containing all instances of imported baitfish described in those documents. We considered three types of import records: quantitative (an instance of live baitfish importation where the quantity of fish was reported); qualitative (the quantity of fish was not reported); or non-existent (the department explicitly confirmed that the records do not exist).

For both qualitative and quantitative import records, we extracted the importing state, year, source facility (e.g., original farm where the fish were produced), source state, and the fish species, though we note that not every record contained every piece of information. For quantitative records only, we also extracted the quantity and unit associated with the import record. We also listed some key pieces of metadata to allow the spreadsheet row to be traced back to an original document, including the document’s file name and the department from which that document was obtained. We did not record information that clearly related to saltwater fish, non-finfish animals, eggs/genetic material, or fish imports for other purposes (e.g., aquarium trade, fish stocking).

We assigned each row to a binomial species name, based on the common name provided in the record. In the few cases where the common name referred to a higher taxonomic rank than species, the row was instead assigned to the appropriate rank (e.g., Castostomidae for “sucker”). We also assigned each row a rating for the likelihood that the record corresponded to the live baitfish trade (likely, possible, unlikely, or insufficient information), on the basis of species, supplier business name, destination business name, and any other information contained in the original document. We restricted subsequent analysis to “likely” rows, enabling us to exercise caution and ensure that the trade flows represented in the data are likely to correspond to the live baitfish trade rather than other purposes. While some vendors sell fish for both bait and ornamental purposes, it was usually clear whether a shipment corresponded to the live baitfish trade rather than ornamental purposes by reviewing the destination business name (e.g., a bait shop, rather than an aquarium store) and the quantity and mix of species included (e.g., several pounds of fathead minnows and golden shiners, rather than a dozen goldfish with other ornamental species). However, in a handful of cases, we exercised our best judgment; this is a limitation of the data source that has also been noted in other sources, particularly for the goldfish market [15].

Since quantities of fish were expressed in different units (e.g., number of fish, pounds, or gallons), we converted all quantitative records to the number of fish. This conversion was made for each species on the basis of published conversion factors or information made publicly available by producers (S1 Table) [15,25,26].

Finally, we conducted an internet search for publicly available documents published by government departments, usually lists of approved live baitfish suppliers for a particular state. These records were parsed and included in the dataset as qualitative records.

## Results

Forty-nine open records requests across 39 states were fulfilled. Of these, 31 provided documents, yielding a total of 3.2 GB of original documents (S1 Fig).

The final dataset contained 7,605 data points. Of these data points, 7,587 corresponded to a specific movement of fish across a state border—5,643 containing information about fish quantities, and 1,962 were qualitative. The remaining 18 data points simply documented the non-existence of the requested data for a particular state. Likewise, 69.9% of data points were assigned to the category of “likely” relating to the live baitfish trade. There was also variation in types of information maintained across states. In some states, the documents contained information on every individual shipment of live baitfish into the state along with species and quantities, allowing rich insight into trade flows involving those states. In other cases, documents gave some qualitative insight into live baitfish origins but without quantity or species identities—this was common for states where regulations only require importing businesses to hold a license, rather than detailed import permits for each shipment.

The quantitative data captured 39.3 million live baitfish, each of which was transported across a U.S. state border during 2022–24. Among records that could be linked with a species name, most common species were fathead minnows (*Pimephales promelas*), golden shiners (*Notemigonus crysoleucas*), goldfish (*Carassius auratus*), alewives (*Alosa pseudoharengus*), and suckers (Catostomidae) (Table 2; for results disaggregated by species and state, see S2 Table). The bulk of these fish originated from Arkansas (64.6%), South Dakota (22.4%), Minnesota (1.3%), and New York (1.2%). 1.1% of live baitfish could not be traced to a state of origin. Likewise, some records originated from Massachusetts; however, as Massachusetts has no in-state live baitfish production [15], these records likely correspond to baitfish that were imported by a Massachusetts-based wholesaler but produced in a third state.

**Table 2:** Live baitfish species represented in obtained documents.

Species	Number of live baitfish (approx)	Percent
*Notemigonus crysoleucas*	23,594,087	60.0%
*Pimephales promelas*	14,598,149	37.1%
*Carassius auratus*	481,370	1.2%
*Alosa pseudoharengus*	465,000	1.2%
*Catostomidae*	63,214	0.2%
*Lepomis macrochirus*	22,820	0.1%
*Centrarchidae*	21,600	0.1%
*Lepomis cyanellus*	19,300	0.0%
*Ictalurus furcatus*	8,300	0.0%
*Ictalurus punctatus*	8,300	0.0%
*Pylodictis olivaris*	8,300	0.0%
*Hudsonius hudsonius*	5,344	0.0%
*Anguilla rostrata*	254	0.0%

The qualitative data reveals the flow of 26 fish species (e.g., goldfish *C. auratus*, bluegill *Lepomis macrochirus*, golden shiners *N. crysoleucas*, fathead minnows *P. promelas*) or taxonomic groups into 20 states (e.g., Illinois, Indiana, South Carolina, South Dakota, West Virginia) from a further 24 states (e.g., Arkansas, Minnesota, Wisconsin) (S4 Table). Moreover, many of these combinations of species and importing states were not present in the quantitative data, indicating that the qualitative data offers additional insights about the flow of a variety of live baitfish species across state borders. One example from the qualitative data is the importation of the invasive species mosquitofish (*Gambusia* sp.) from a major bait producer in Arkansas into Ohio under a bait dealer’s license; this example is noteworthy as the proximity of mosquitofish to the production of major live baitfish species may pose a risk of contamination as occurred in Vermont in 2018 [27].

**Fig 1:**
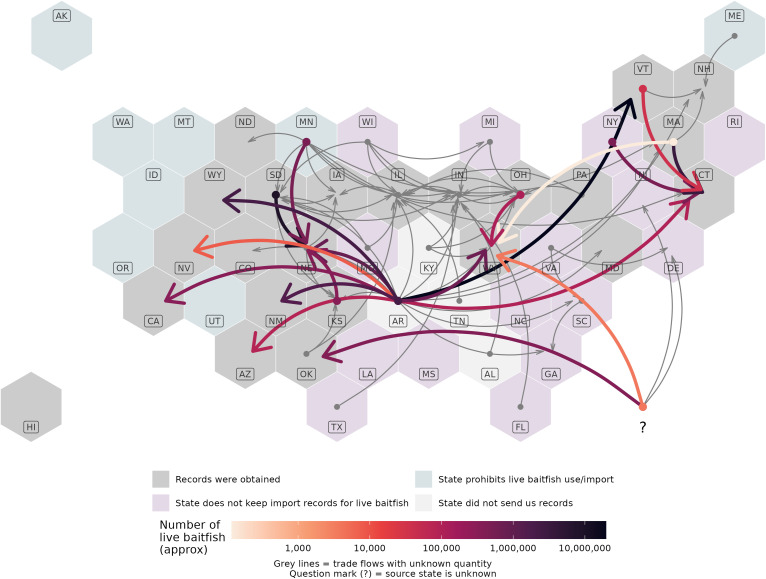
Hexagon grid map illustrating the transport of live baitfish across state borders. Arrows denote trade flows from a state of origin (circle) to a destination state (arrowhead). Thin grey arrows denote trade flows whose quantity could not be determined. Question mark denotes trade flows whose state of origin is unknown. States are colored pale blue if they do not permit the importation or use of live baitfish, pink if they reported that baitfish importation records were non-existent, white if they did not send us records, and grey in all other cases. Base map by Andrew Hill (https://team.carto.com/u/andrew/tables/andrew.us_states_hexgrid/public/map).

Visualizing the trade flows of live baitfish spatially reveals several patterns. The map in Fig 1 shows the aggregated trade flows for each pair of exporting and importing states; for quantitative trade flows, the total number of fish traded between each pair of states is visualized using colored arrows, and qualitative trade flows are also visualized using grey arrows (this is the same data as S2 Table, except aggregated by species). Arkansas stands out as a source state, having shipped live baitfish to at least 20 states across the country; some of these states only imported baitfish from Arkansas. However, many states imported live baitfish from multiple states. Minnesota, Ohio, Wisconsin, and South Dakota were also the origin of many trade flows. Some states were both the origin and destination for live baitfish, which could indicate the re-export of live baitfish by wholesalers (e.g., Vermont).

## Discussion

In this study, we have examined a new source of data that provides insight into a pathway by which millions of minnows are transported long distances across the United States. Using original documents obtained from governments across the country using freedom-of-information/open records requests, we have provided key information into the transport of live baitfish across state borders, which can enable policymakers and other stakeholders to more rigorously understand the risks posed by this pathway of aquatic invasive species and pathogens [19,20,22,28–35]. By identifying high-traffic trade routes and quantifying species data, this dataset provides actionable insights for state agencies to strengthen biosecurity and environmental policy. Our dataset is the first that contains quantitative estimates of live baitfish trade flows across the United States since Meronek [36]’s analysis of imports in the North Central region, over 30 years ago.

Our findings offer support to the general conclusions drawn from previous studies in the United States. Most notably, Gunderson [5]’s survey of industry representatives from the baitfish supply chain in the Mississippi River Basin found that states import live baitfish most frequently from a few key states, namely Arkansas, Minnesota, South Dakota, and Wisconsin—these states also appeared in our analysis as the origin of live baitfish imported by states across the country. The finding that Minnesota, Ohio, Wisconsin, and South Dakota were the origin of many trade flows is consistent with those states’ high production quantities reported in the U.S. Census of Aquaculture [15]. Van Senten and Engle [14] found that baitfish and sportfish producers in Arkansas, Alabama, Wisconsin, and other states make shipments to numerous destination states, a finding that is reflected in our own map of trade flows. Our results are consistent with Meronek [36]’s finding that Illinois imports live baitfish in significant quantities, though the governments of Michigan and Ohio—two other states examined by Meronek that imported live baitfish—reported to us that they do not keep records on live baitfish imports. Our finding that many states import live baitfish from several origin states is also consistent with previous observations [23].

Beyond this general agreement, our dataset offers several key advances. Most importantly, many of the trade flows represented in our dataset were accompanied by an estimated number of live baitfish, enabling policymakers and other stakeholders to gauge the relative quantity of imports from various states. For example, our results indicate that West Virginia imported the majority of its live baitfish (approximately 670,000 fish) from Arkansas during the study period, with most of the remainder (110,000) from Ohio. Another advance is that our dataset allows trade flows to be disaggregated by species. To illustrate, Nebraska’s live baitfish imports during the study period were predominantly fathead minnow (approximately 9.3 million fish), golden shiner (710,000), or goldfish (310,000), with smaller contributions from other species. The dataset also enables the examination of trends in importing states.

Many of the importing states (e.g., Wyoming, Nevada, and California) had trade flows originating from only one state. Other states (e.g., Connecticut, Nebraska, and West Virginia) had trade flows originating from multiple states. Also notable were states that both imported and exported live baitfish (e.g., Vermont, Illinois, and Indiana). Illinois and Indiana had numerous import and export connections with other states. These two states may be importing live baitfish from numerous states and exporting to other numerous states — however, these two states had only qualitative data, so it could be the case that only a couple of these numerous connections corresponded to real trade flows of any meaningful quantity. The former case would bring important implications for invasive species and disease risk [37]. These apparent trade hubs may represent individual shipments of live baitfish being shipped to a wholesale distributor in an intermediate state before being re-exported to a final destination [38], though it is also possible that some states imported baitfish at one time of year and exported baitfish at other times due to seasonal fluctuations in supply and demand [39,40]. This level of detail allows policymakers and other stakeholders to assess the risk posed by the long-distance trade of live baitfish, including risks of aquatic invasive species and pathogens, in greater detail than has previously been possible.

Zooming out to consider our study in a global context, our results contribute to the body of evidence on the role that transport plays in the spread of aquatic invasive species via the live baitfish trade. Kalous et al [8] examined the live baitfish market in Prague, Czechia, finding that the invasive species Prussian carp (*Carassius gibelio*) and topmouth gudgeon (*Pseudorasbora parva*) are sold as live baitfish and that over one-third of anglers are travelling over 50 km from Prague, representing a large range relative to this small European country. Likewise, Drăgan et al [41] found that baitfish minnows (minnows *Phoxinus* sp. and stone loach *Barbatula barbatula*) had colonized 12 of 25 alpine lakes within Retezat National Park, Romania, likely due to transport associated with fishing activity. Studies around the world have also linked the transport of bait with the spread of invertebrates, as seen in the spread of redclaw crayfish (*Cherax quadricarinatus*) in Indonesia and polychaete worms in the Netherlands, Portugal, and the Azores islands [7,42,43]. The present study complements this existing body of evidence by providing insight into the role of the live baitfish trade in the context of interstate shipments as part of a large, established industry.

A limitation of our study was the variation in the consistency of documents held by state governments. To illustrate, our quantitative data described 39.3 million live baitfish between 2022–24, in addition to baitfish transported without quantity records; compare this to the annual production in the United States, which exceeds one billion baitfish [15]. Much of the country’s production of “baitfish” ends up being used for pond stocking, feeder fish, and other purposes [44]. However, the main reason for the discrepancy is that many state governments reported that they do not hold any records on the live baitfish transported into their states, including states where the impacts of aquatic invasive species have been significant (e.g., Michigan). The variation in data quality is consistent with previous research that found variation in U.S. states’ spending on invasive species management as a whole [45]. We also note that, in a few cases, live baitfish appeared to be imported for re-export. While several states in our dataset had both imports and exports, the only state where both imports and exports had quantity records was Vermont. Vermont’s quantitative re-exports were small with an estimated 35,000 fish, which equates to 0.2% of Vermont’s estimated 19.5 million fish imported, indicating that re-exports would not significantly inflate the estimate of fish traded in our dataset.

Moreover, many state governments did provide documents, but these documents did not contain sufficient information to accurately quantify trade flows. Some states grant umbrella import permits, making it difficult to measure the total size of the trade flow in those states (e.g., Oklahoma). Other states collect information on the business responsible for importation, but not the original source of the fish. This means that several trade flows could not be traced back to where the baitfish were farmed, though a record from Massachusetts did describe a wholesaler from Vermont importing fish that ultimately originated from Arkansas. In other cases, individual licenses are granted on the basis of broad information—one example was an import license granted by West Virginia, with the species listed as “game fish, catfish, minnows, ornamental” and the source listed as seven different fish farms or distributors. This means that, despite the insights offered by our dataset, there are several states where data quality could be further harmonized to support biosurveillance and risk reduction.

There may also be variation in risk depending on whether imported live baitfish are sourced from farms *vs.* wild sources in their state of origin [46]. Gunderson [5] suggests that for the baitfish species traded in the largest quantities — fathead minnows, golden shiners, and goldfish — exports overwhelmingly originate from farms, and wild-caught baitfish may be used in-state but are exported rarely. Wild-caught fish are usually self-collected by anglers or collected for sale in local bait shops, and some states prohibit the export of wild-caught baitfish (e.g., Michigan) [22,33]. That said, Purdy [47] notes that wild-caught fish may be exported to meet demand when farm-raised fish are insufficient to meet supply. Empirical studies have detected no statistically significant differences in overall viral or pathogen loads between farmed *vs.* wild-caught live baitfish, though there may be some differences for specific pathogens or particular host species [47,48]. Furthermore, many farm-raised stocks are sourced from wild populations, meaning that the distinction between farm-raised *vs.* wild-caught baitfish is a blurry one [29].

There are several ways in which the results from this study can be applied to environmental policy. Firstly, the study identified specific high-traffic routes, such as the flow of fish from Arkansas to at least 20 states. State fish and wildlife departments can use this data to prioritize inspections at the specific border crossings or retail hubs that sit along these major trade arteries [49,50]. Secondly, since the study quantified the number of fish and identified the most common species, epidemiological studies can use this data to improve the accuracy of models for the spread of fish diseases like viral hemorrhagic septicemia. Thirdly, policymakers in states with limited records can use these findings as a justification to modernize their record-keeping. By adopting the quantitative tracking methods used by the “data-rich” states identified in the study, other states can better protect their local ecosystems from unmonitored imports. Fourthly, the study suggests that states like Massachusetts may act as wholesalers—importing fish only to re-export them to third states. This reveals a secondary market that is important for biosecurity. The study’s results could be used to include wholesaler audits rather than just producer audits in management plans, ensuring that fish remain traceable as they move through these transit hubs [51].

Future research could further improve data quality by collecting data using different methods. A fruitful endeavor would be to survey local bait shops and supply chain actors conducting business in the states that we have identified as having gaps in live baitfish import data [5,14,23]. Such surveys could include questions about shipments across state borders and the quantitative scale of these shipments, which would help to fill in the gaps in the data. Likewise, a dedicated risk assessment that identifies the aquatic invasive species most likely to spread into particular geographies via the live baitfish trade, perhaps with an anticipated estimate of the economic damage of such an invasion, could provide further insight for decision-makers seeking to balance policy goals.

While this study has focused on state-level data, one could also consider the influence of Federal-level regulations and permits on the interstate trade of live baitfish. There are several Federal regulations that may restrict or limit the trade of live baitfish. Most notably, the Lacey Act empowers the U.S. Fish and Wildlife Service (FWS) to list species as “injurious wildlife” and restrict trade in those species [52,53]. While the courts in 2017 found that the Lacey Act does not grant the FWS power to prohibit interstate shipment in most cases, there have been several efforts in Congress to explicitly grant the FWS this power. The Environmental Protection Agency has the power to regulate escaped species from aquaculture facilities, and the National Park Service prohibits the use of live baitfish in many national parks [52,53]; each of these may have implications for the interstate movement of fish species. The U.S. Department of Agriculture and the Centers for Disease Control and Prevention can also regulate and inspect imports into the U.S. from abroad, though this is less relevant for interstate trade [52]. Future research could examine whether these Federal regulations have any impact on the interstate trade of live baitfish species.

On the other extreme, the complexity of interstate trade is further compounded by within-state movements. Interstate records capture the initial entry of fish into a state, but subsequent transport from wholesalers to retail bait shops or between different watersheds within a state creates further movement [54]. These internal flows present both a risk of waterbody-level species spread and an opportunity for risk reduction. For instance, states that implement “zone-based” regulations—restricting the movement of live bait between specific water bodies or across drainage divides—could help to contain hitchhiking species or pathogens even if those species have already crossed state lines [18]. Future research could support these efforts by mapping the movement and behavior of anglers within states to better understand the risks of invasive species transport at the level of local waterbodies [55,56].

Aquatic invasive species and pathogens pose risks to recreational fishing, tourism, commercial fisheries, water-based industries, and local biodiversity [12,13,57–59]. To protect fishing opportunities and other ecosystem functions, several states have implemented restrictions on the importation of live baitfish from other states (e.g., Maine, Minnesota, Montana) and/or spatial restrictions on the use of live baitfish in ecologically sensitive areas (e.g., Colorado, Maine, Nevada) [18]. Despite high-quality data in some states, other state governments hold limited records or even no records on the flow of live baitfish into the state. This suggests that state-level data collection could be further harmonized to support biosecurity monitoring and regulation. The dataset highlights specific opportunities for strengthening state monitoring and regulations concerning the interstate live baitfish trade flows.

## Supporting information

S1 Text Open records request letter. (DOCX)

S1 Table Biological unit conversion factors. (DOCX)

S1 Figure Flowchart of freedom-of-information requests and trade data. (PNG)

S2 Table Trade flows disaggregated by species and state. (DOCX)
