# Supplementary material for: Live fish highway: Uncovering the pathways that move millions of minnows across the United States
Source: PLoS One. 2026 May 13;21(5):e0347150. doi: 10.1371/journal.pone.0347150 (PMC13170828; doi:10.1371/journal.pone.0347150)
Supplement: S1 Text — (DOCX) [file pone.0347150.s001.docx]

**S1 Text. Open records request letter.**

The freedom-of-information/open records requests were worded as follows. Minor adjustments were made depending on state-specific regulations.

I am writing with a request for public data/records under the state's open record legislation. Specifically, I would like to request the following records relating to the state's live baitfish industry:

*[If the department is responsible for governing baitfish imports:]*

- Copies of the completed license application documents corresponding to all licenses granted for the import of live finfish into the state during calendar years 2022, 2023, and 2024.

- Any additional documents held by the department that contains details of species and quantity of live fish imported into the state during that period (unless such information is already included in above dot point)

Notes on the above request:

- If the department keeps record of the purpose of individual imports (i.e. the intended end-use for a specific shipment or batch of fish), I am happy to receive *only* the documents that relate to imports of finfish for sale as bait or use as bait. However, if the department does not keep records about the purpose of individual imports/records, then I would like all records relating to live finfish imports into the state.

- The request is for live fish only.

- I am interested in both freshwater and/or saltwater finfish, to the extent of the records held by the department.

*[If the department is responsible for governing in-state baitfish production:]*

- Number of aquaculture farms in the state that produce baitfish

- For each farm, the record describing the species and quantity (tonnage, dozen, or dollar value) of baitfish produced in the most recent time period (e.g. year) for which data exists

Note: I am aware of the existence of the U.S. aquaculture census, but I nevertheless require this data from the department to ensure that I have the most recent and comprehensive data possible.
