# Supplementary material for: Live fish highway: Uncovering the pathways that move millions of minnows across the United States
Source: PLoS One. 2026 May 13;21(5):e0347150. doi: 10.1371/journal.pone.0347150 (PMC13170828; doi:10.1371/journal.pone.0347150)
Supplement: S1 Table — (DOCX) [file pone.0347150.s002.docx]

**S1 Table**. Biological unit conversion factors.

| **Unit of fish quantity** | **Species (binomial)** | **Number of fish per unit quantity** | **Source** |
| --- | --- | --- | --- |
| pounds | *Pimephales promelas* | 196.8 | United States Department of Agriculture. *2022 Census of Agriculture: 2023 Census of Aquaculture*. (2024). |
| gallons | *Pimephales promelas* | 1574.4 | Meronek, T. G., Copes, F. A. & Coble, D. W. *The Bait Industry in Illinois, Michigan, Minnesota, Ohio, South Dakota, and Wisconsin*. https://www.ncrac.org/files/inline-files/tb105_0.pdf (1997). |
| pounds | *Notemigonus crysoleucas* | 134 | United States Department of Agriculture. *2022 Census of Agriculture: 2023 Census of Aquaculture*. (2024). |
| gallons | *Notemigonus crysoleucas* | 1072 | Meronek, T. G., Copes, F. A. & Coble, D. W. *The Bait Industry in Illinois, Michigan, Minnesota, Ohio, South Dakota, and Wisconsin*. https://www.ncrac.org/files/inline-files/tb105_0.pdf (1997). |
| pounds | *Carassius auratus* | 77.7 | United States Department of Agriculture. *2022 Census of Agriculture: 2023 Census of Aquaculture*. (2024). |
| pounds | *Lepomis macrochirus* | 50 | Nemo Bait & Fisheries. Species & Information. *Nemo Bait & Fisheries* https://web.archive.org/web/20251010013625/http://nemofisheries.com/species--information.html. |
| pounds | *Catostomidae* | 40.6 | United States Department of Agriculture. *2022 Census of Agriculture: 2023 Census of Aquaculture*. (2024). |
| gallons | *Catostomidae* | 324.8 | Meronek, T. G., Copes, F. A. & Coble, D. W. *The Bait Industry in Illinois, Michigan, Minnesota, Ohio, South Dakota, and Wisconsin*. https://www.ncrac.org/files/inline-files/tb105_0.pdf (1997). |
| gallons | *Carassius auratus* | 621.6 | Meronek, T. G., Copes, F. A. & Coble, D. W. *The Bait Industry in Illinois, Michigan, Minnesota, Ohio, South Dakota, and Wisconsin*. https://www.ncrac.org/files/inline-files/tb105_0.pdf (1997). |
| pounds | *Ictalurus punctatus* | 10 | Nemo Bait & Fisheries. Species & Information. *Nemo Bait & Fisheries* https://web.archive.org/web/20251010013625/http://nemofisheries.com/species--information.html. |
| pounds | *Ictalurus furcatus* | 10 | Assumed comparable to *I. punctatus* |
| pounds | *Pylodictis olivaris* | 10 | Assumed comparable to *I. punctatus* |
| gallons | *Centrarchidae* | 400 | Nemo Bait & Fisheries. Species & Information. *Nemo Bait & Fisheries* https://web.archive.org/web/20251010013625/http://nemofisheries.com/species--information.html.  Meronek, T. G., Copes, F. A. & Coble, D. W. *The Bait Industry in Illinois, Michigan, Minnesota, Ohio, South Dakota, and Wisconsin*. https://www.ncrac.org/files/inline-files/tb105_0.pdf (1997). |
| pounds | *Lepomis cyanellus* | 50 | Nemo Bait & Fisheries. Species & Information. *Nemo Bait & Fisheries* https://web.archive.org/web/20251010013625/http://nemofisheries.com/species--information.html.  Assumed comparable to related bluegill and sunfish species |
