# Supplementary figures and images for: Live fish highway: Uncovering the pathways that move millions of minnows across the United States
Source: PLoS One. 2026 May 13;21(5):e0347150. doi: 10.1371/journal.pone.0347150 (PMC13170828; doi:10.1371/journal.pone.0347150)

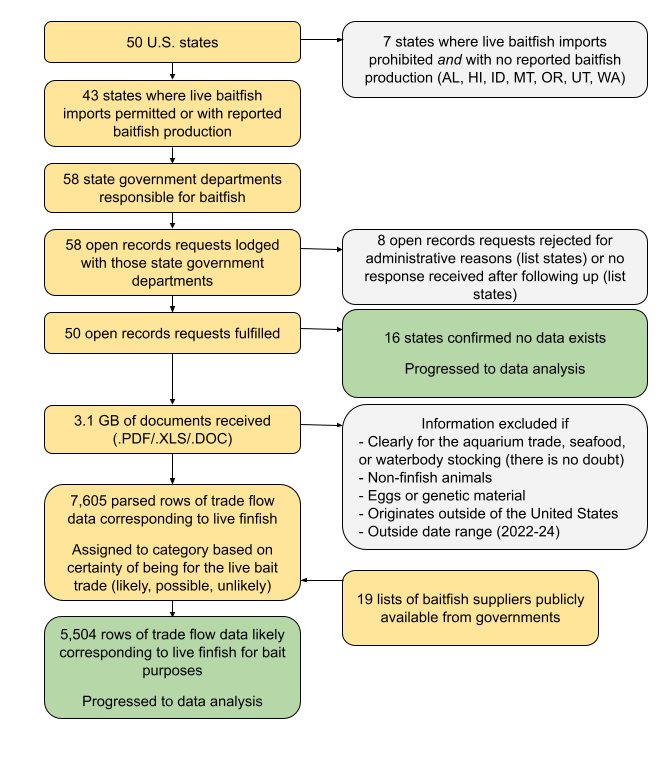

Supplement: S1 Figure — (PNG) [file pone.0347150.s003.png]
