# Supplementary material for: Live fish highway: Uncovering the pathways that move millions of minnows across the United States
Source: PLoS One. 2026 May 13;21(5):e0347150. doi: 10.1371/journal.pone.0347150 (PMC13170828; doi:10.1371/journal.pone.0347150)
Supplement: S2 Table — (DOCX) [file pone.0347150.s004.docx]

**S2 Table**. Trade flows disaggregated by species and state.

| **Species** | **Destination state** | **Source state** | **Number of live baitfish (approx)** |
| --- | --- | --- | --- |
| *Notemigonus crysoleucas* | Vermont | Arkansas | 19,263,438 |
| *Pimephales promelas* | Nebraska | South Dakota | 8,759,568 |
| *Pimephales promelas* | Wyoming | Arkansas | 2,212,032 |
| *Notemigonus crysoleucas* | Connecticut | Massachusetts | 1,917,340 |
| *Pimephales promelas* | Connecticut | Massachusetts | 1,289,040 |
| *Pimephales promelas* | New Mexico | Arkansas | 775,195 |
| *Notemigonus crysoleucas* | New Mexico | Arkansas | 707,386 |
| *Alosa pseudoharengus* | Connecticut | New York | 465,000 |
| *Notemigonus crysoleucas* | Nebraska | Arkansas | 448,096 |
| *Pimephales promelas* | Nebraska | Minnesota | 367,622 |
| *Pimephales promelas* | West Virginia | Arkansas | 270,238 |
| *Notemigonus crysoleucas* | Nebraska | Kansas | 258,218 |
| *Notemigonus crysoleucas* | Oklahoma | State unknown | 252,992 |
| *Notemigonus crysoleucas* | West Virginia | Arkansas | 226,316 |
| *Pimephales promelas* | Vermont | Arkansas | 203,568 |
| *Pimephales promelas* | Nebraska | Arkansas | 186,026 |
| *Carassius auratus* | Nebraska | Arkansas | 182,672 |
| *Notemigonus crysoleucas* | California | Arkansas | 156,000 |
| *Pimephales promelas* | California | Arkansas | 156,000 |
| *Carassius auratus* | West Virginia | Arkansas | 124,299 |
| *Carassius auratus* | Nebraska | Minnesota | 105,672 |
| *Pimephales promelas* | Oklahoma | State unknown | 89,860 |
| *Pimephales promelas* | West Virginia | Ohio | 76,948 |
| *Pimephales promelas* | Arizona | Arkansas | 76,250 |
| *Pimephales promelas* | Connecticut | Arkansas | 66,720 |
| *Catostomidae* | Nebraska | South Dakota | 59,113 |
| *Notemigonus crysoleucas* | Connecticut | Vermont | 35,108 |
| *Pimephales promelas* | Nebraska | Kansas | 27,552 |
| *Carassius auratus* | Oklahoma | State unknown | 27,195 |
| *Notemigonus crysoleucas* | Connecticut | Arkansas | 25,967 |
| *Notemigonus crysoleucas* | West Virginia | Ohio | 25,862 |
| *Carassius auratus* | Nebraska | Kansas | 24,320 |
| *Centrarchidae* | Nebraska | Minnesota | 21,600 |
| *Lepomis macrochirus* | West Virginia | Arkansas | 20,550 |
| *Lepomis cyanellus* | Nebraska | Kansas | 19,300 |
| *Carassius auratus* | West Virginia | Ohio | 8,935 |
| *Ictalurus furcatus* | West Virginia | Arkansas | 8,300 |
| *Ictalurus punctatus* | West Virginia | Arkansas | 8,300 |
| *Pylodictis olivaris* | West Virginia | Arkansas | 8,300 |
| *Carassius auratus* | Arizona | Arkansas | 7,100 |
| *Hudsonius hudsonius* | Connecticut | Arkansas | 5,344 |
| *Pimephales promelas* | Nevada | Arkansas | 4,136 |
| *Catostomidae* | Nebraska | Kansas | 4,100 |
| *Notemigonus crysoleucas* | Nevada | Arkansas | 3,300 |
| *Notemigonus crysoleucas* | Arizona | Arkansas | 3,250 |
| *Notemigonus crysoleucas* | Nebraska | Minnesota | 3,216 |
| *Notemigonus crysoleucas* | Nebraska | South Dakota | 3,216 |
| *Pimephales promelas* | West Virginia | State unknown | 2,164 |
| *Notemigonus crysoleucas* | West Virginia | State unknown | 938 |
| *Lepomis macrochirus* | Wyoming | Arkansas | 760 |
| *Carassius auratus* | West Virginia | State unknown | 621 |
| *Lepomis macrochirus* | Nebraska | Arkansas | 610 |
| *Carassius auratus* | Nevada | Arkansas | 553 |
| *Lepomis macrochirus* | Oklahoma | State unknown | 500 |
| *Lepomis macrochirus* | New Mexico | Arkansas | 400 |
| *Anguilla rostrata* | Connecticut | Massachusetts | 134 |
| *Anguilla rostrata* | West Virginia | Massachusetts | 120 |
| *Pimephales promelas* | Massachusetts | Arkansas | Quantity unknown (but non-zero) |
| *Notemigonus crysoleucas* | Massachusetts | Arkansas | Quantity unknown (but non-zero) |
| *Species unknown* | Illinois | Arkansas | Quantity unknown (but non-zero) |
| *Species unknown* | Kansas | Arkansas | Quantity unknown (but non-zero) |
| *Species unknown* | Kansas | South Dakota | Quantity unknown (but non-zero) |
| *Pimephales promelas* | West Virginia | Kentucky | Quantity unknown (but non-zero) |
| *Species unknown* | Kansas | Oklahoma | Quantity unknown (but non-zero) |
| *Pimephales promelas* | South Carolina | Arkansas | Quantity unknown (but non-zero) |
| *Pimephales promelas* | South Carolina | Virginia | Quantity unknown (but non-zero) |
| *Pimephales promelas* | South Dakota | Arkansas | Quantity unknown (but non-zero) |
| *Carassius auratus* | Illinois | Ohio | Quantity unknown (but non-zero) |
| *Carassius auratus* | Illinois | Missouri | Quantity unknown (but non-zero) |
| *Pimephales promelas* | Colorado | Arkansas | Quantity unknown (but non-zero) |
| *Notemigonus crysoleucas* | South Dakota | Arkansas | Quantity unknown (but non-zero) |
| *Notemigonus crysoleucas* | West Virginia | Kentucky | Quantity unknown (but non-zero) |
| *Species unknown* | Indiana | Arkansas | Quantity unknown (but non-zero) |
| *Notemigonus crysoleucas* | Illinois | Oklahoma | Quantity unknown (but non-zero) |
| *Species unknown* | Indiana | Wisconsin | Quantity unknown (but non-zero) |
| *Notemigonus crysoleucas* | Illinois | Arkansas | Quantity unknown (but non-zero) |
| *Species unknown* | Iowa | Minnesota | Quantity unknown (but non-zero) |
| *Species unknown* | Iowa | South Dakota | Quantity unknown (but non-zero) |
| *Species unknown* | Illinois | Missouri | Quantity unknown (but non-zero) |
| *Species unknown* | Indiana | Ohio | Quantity unknown (but non-zero) |
| *Notemigonus crysoleucas* | Illinois | Tennessee | Quantity unknown (but non-zero) |
| *Pimephales promelas* | Illinois | Arkansas | Quantity unknown (but non-zero) |
| *Anguilla rostrata* | West Virginia | Kentucky | Quantity unknown (but non-zero) |
| *Campostoma sp.* | West Virginia | Kentucky | Quantity unknown (but non-zero) |
| *Lepomis macrochirus* | West Virginia | Kentucky | Quantity unknown (but non-zero) |
| *Lepomis macrochirus* | South Carolina | Arkansas | Quantity unknown (but non-zero) |
| *Lepomis macrochirus* | Illinois | Missouri | Quantity unknown (but non-zero) |
| *Lepomis macrochirus* | Illinois | Arkansas | Quantity unknown (but non-zero) |
| *Lepomis macrochirus* | Illinois | Tennessee | Quantity unknown (but non-zero) |
| *Pimephales promelas* | Illinois | Indiana | Quantity unknown (but non-zero) |
| *Catostomus commersonii* | North Dakota | Minnesota | Quantity unknown (but non-zero) |
| *Pimephales promelas* | Illinois | Missouri | Quantity unknown (but non-zero) |
| *Pimephales promelas* | Illinois | Wisconsin | Quantity unknown (but non-zero) |
| *Catostomus commersonii* | South Dakota | Minnesota | Quantity unknown (but non-zero) |
| *Notemigonus crysoleucas* | Illinois | Ohio | Quantity unknown (but non-zero) |
| *Catostomidae* | North Dakota | Minnesota | Quantity unknown (but non-zero) |
| *Notemigonus crysoleucas* | Illinois | Indiana | Quantity unknown (but non-zero) |
| *Notemigonus crysoleucas* | South Carolina | Arkansas | Quantity unknown (but non-zero) |
| *Pimephales promelas* | South Dakota | Wisconsin | Quantity unknown (but non-zero) |
| *Notemigonus crysoleucas* | South Carolina | Virginia | Quantity unknown (but non-zero) |
| *Carassius auratus* | West Virginia | Kentucky | Quantity unknown (but non-zero) |
| *Semotilus sp.* | Ohio | Pennsylvania | Quantity unknown (but non-zero) |
| *Pimephales vigilax* | Ohio | Kentucky | Quantity unknown (but non-zero) |
| *Pimephales promelas* | Ohio | Kentucky | Quantity unknown (but non-zero) |
| *Notemigonus crysoleucas* | Illinois | Missouri | Quantity unknown (but non-zero) |
| *Pomoxis sp.* | Ohio | Arkansas | Quantity unknown (but non-zero) |
| *Lepomis microlophus* | Ohio | Arkansas | Quantity unknown (but non-zero) |
| *Pimephales promelas* | Ohio | Arkansas | Quantity unknown (but non-zero) |
| *Carassius auratus* | Illinois | Pennsylvania | Quantity unknown (but non-zero) |
| *Species unknown* | Georgia | South Carolina | Quantity unknown (but non-zero) |
| *Species unknown* | Indiana | Illinois | Quantity unknown (but non-zero) |
| *Species unknown* | Indiana | Michigan | Quantity unknown (but non-zero) |
| *Species unknown* | Indiana | Alabama | Quantity unknown (but non-zero) |
| *Species unknown* | Illinois | Wisconsin | Quantity unknown (but non-zero) |
| *Species unknown* | Illinois | South Dakota | Quantity unknown (but non-zero) |
| *Carassius auratus* | Illinois | Arkansas | Quantity unknown (but non-zero) |
| *Species unknown* | Iowa | Nebraska | Quantity unknown (but non-zero) |
| *Pimephales promelas* | Illinois | Tennessee | Quantity unknown (but non-zero) |
| *Species unknown* | West Virginia | Kentucky | Quantity unknown (but non-zero) |
| *Fundulus grandis* | Delaware | State unknown | Quantity unknown (but non-zero) |
| *Species unknown* | Indiana | Texas | Quantity unknown (but non-zero) |
| *Notemigonus crysoleucas* | South Dakota | Wisconsin | Quantity unknown (but non-zero) |
| *Cyprinus sp.* | West Virginia | Kentucky | Quantity unknown (but non-zero) |
| *Ictalurus punctatus* | West Virginia | Kentucky | Quantity unknown (but non-zero) |
| *Ameiurus sp.* | West Virginia | Kentucky | Quantity unknown (but non-zero) |
| *Notemigonus crysoleucas* | Ohio | Arkansas | Quantity unknown (but non-zero) |
| *Notemigonus crysoleucas* | Ohio | Indiana | Quantity unknown (but non-zero) |
| *Species unknown* | Ohio | Indiana | Quantity unknown (but non-zero) |
| *Species unknown* | Ohio | Minnesota | Quantity unknown (but non-zero) |
| *Species unknown* | Ohio | Arkansas | Quantity unknown (but non-zero) |
| *Lepomis macrochirus* | Ohio | Kentucky | Quantity unknown (but non-zero) |
| *Notropis atherinoides* | Ohio | Kentucky | Quantity unknown (but non-zero) |
| *Notemigonus crysoleucas* | Illinois | Wisconsin | Quantity unknown (but non-zero) |
| *Pimephales promelas* | Illinois | Ohio | Quantity unknown (but non-zero) |
| *Notemigonus crysoleucas* | Delaware | Virginia | Quantity unknown (but non-zero) |
| *Notemigonus crysoleucas* | Colorado | Arkansas | Quantity unknown (but non-zero) |
| *Pimephales promelas* | Illinois | Pennsylvania | Quantity unknown (but non-zero) |
| *Notemigonus crysoleucas* | Illinois | Pennsylvania | Quantity unknown (but non-zero) |
| *Pimephales promelas* | South Dakota | Missouri | Quantity unknown (but non-zero) |
| *Catostomus commersonii* | Ohio | Wisconsin | Quantity unknown (but non-zero) |
| *Pimephales vigilax* | Ohio | Minnesota | Quantity unknown (but non-zero) |
| *Cyprinidae* | Ohio | Kentucky | Quantity unknown (but non-zero) |
| *Erimyzon oblongus* | Ohio | Kentucky | Quantity unknown (but non-zero) |
| *Species unknown* | Ohio | Wisconsin | Quantity unknown (but non-zero) |
| *Gambusia sp.* | Ohio | Arkansas | Quantity unknown (but non-zero) |
| *Species unknown* | Iowa | Illinois | Quantity unknown (but non-zero) |
| *Species unknown* | Ohio | Michigan | Quantity unknown (but non-zero) |
| *Notemigonus crysoleucas* | South Dakota | Missouri | Quantity unknown (but non-zero) |
| *Species unknown* | New Hampshire | Vermont | Quantity unknown (but non-zero) |
| *Species unknown* | New Hampshire | Massachusetts | Quantity unknown (but non-zero) |
| *Fundulus grandis* | Delaware | Virginia | Quantity unknown (but non-zero) |
| *Species unknown* | Michigan | Wisconsin | Quantity unknown (but non-zero) |
| *Species unknown* | Indiana | Florida | Quantity unknown (but non-zero) |
| *Carassius auratus* | Illinois | Oklahoma | Quantity unknown (but non-zero) |
| *Species unknown* | Massachusetts | Minnesota | Quantity unknown (but non-zero) |
| *Species unknown* | Georgia | Arkansas | Quantity unknown (but non-zero) |
| *Species unknown* | Indiana | Kentucky | Quantity unknown (but non-zero) |
| *Lepomis macrochirus* | Illinois | Wisconsin | Quantity unknown (but non-zero) |
| *Species unknown* | Ohio | Kentucky | Quantity unknown (but non-zero) |
| *Lepomis macrochirus* | Colorado | Arkansas | Quantity unknown (but non-zero) |
| *Notropis atherinoides* | Ohio | Indiana | Quantity unknown (but non-zero) |
| *Notropis atherinoides* | Ohio | Wisconsin | Quantity unknown (but non-zero) |
| *Semotilus sp.* | South Dakota | Wisconsin | Quantity unknown (but non-zero) |
| *Sander vitreus* | South Dakota | Minnesota | Quantity unknown (but non-zero) |
| *Carassius auratus* | Ohio | Kentucky | Quantity unknown (but non-zero) |
| *Alosidae* | Ohio | Kentucky | Quantity unknown (but non-zero) |
| *Species unknown* | Kansas | Nebraska | Quantity unknown (but non-zero) |
| *Pimephales promelas* | Ohio | Wisconsin | Quantity unknown (but non-zero) |
| *Notemigonus crysoleucas* | Ohio | Pennsylvania | Quantity unknown (but non-zero) |
| *Notemigonus crysoleucas* | Ohio | Wisconsin | Quantity unknown (but non-zero) |
| *Semotilus sp.* | Ohio | Wisconsin | Quantity unknown (but non-zero) |
| *Lepomis macrochirus* | Illinois | Indiana | Quantity unknown (but non-zero) |
| *Lepomis macrochirus* | Illinois | Pennsylvania | Quantity unknown (but non-zero) |
| *Carassius auratus* | South Carolina | Arkansas | Quantity unknown (but non-zero) |
| *Lepomis macrochirus* | Ohio | Arkansas | Quantity unknown (but non-zero) |
| *Carassius auratus* | Ohio | Arkansas | Quantity unknown (but non-zero) |
| *Lepomis macrochirus* | South Carolina | Virginia | Quantity unknown (but non-zero) |
| *Notropis atherinoides* | Ohio | Michigan | Quantity unknown (but non-zero) |
| *Species unknown* | Maryland | Arkansas | Quantity unknown (but non-zero) |
| *Species unknown* | New Hampshire | Arkansas | Quantity unknown (but non-zero) |
| *Pimephales promelas* | Illinois | Oklahoma | Quantity unknown (but non-zero) |
| *Centrarchidae* | Ohio | Arkansas | Quantity unknown (but non-zero) |
| *Catostomus commersonii* | Ohio | Pennsylvania | Quantity unknown (but non-zero) |
| *Lepomis macrochirus* | Illinois | Oklahoma | Quantity unknown (but non-zero) |
| *Species unknown* | New Hampshire | Maine | Quantity unknown (but non-zero) |
| *Carassius auratus* | Ohio | Wisconsin | Quantity unknown (but non-zero) |
| *Species unknown* | Connecticut | Minnesota | Quantity unknown (but non-zero) |
| *Centrarchidae* | Ohio | Kentucky | Quantity unknown (but non-zero) |
| *Catostomus commersonii* | South Dakota | Wisconsin | Quantity unknown (but non-zero) |
| *Species unknown* | New Jersey | State unknown | Quantity unknown (but non-zero) |
| *Species unknown* | Indiana | South Dakota | Quantity unknown (but non-zero) |
| *Species unknown* | Ohio | Pennsylvania | Quantity unknown (but non-zero) |
| *Carassius auratus* | Ohio | Indiana | Quantity unknown (but non-zero) |
| *Species unknown* | Indiana | Minnesota | Quantity unknown (but non-zero) |
| *Notropis atherinoides* | Ohio | Pennsylvania | Quantity unknown (but non-zero) |
| *Lepomis macrochirus* | Illinois | Ohio | Quantity unknown (but non-zero) |
